# Entorhinal‐based path integration selectively predicts midlife risk of Alzheimer's disease

**DOI:** 10.1002/alz.13733

**Published:** 2024-02-29

**Authors:** Coco Newton, Marianna Pope, Catarina Rua, Richard Henson, Zilong Ji, Neil Burgess, Christopher T. Rodgers, Matthias Stangl, Maria‐Eleni Dounavi, Andrea Castegnaro, Ivan Koychev, Paresh Malhotra, Thomas Wolbers, Karen Ritchie, Craig W. Ritchie, John O'Brien, Li Su, Dennis Chan, for the PREVENT Dementia Research Programme

**Affiliations:** ^1^ Department of Psychiatry University of Cambridge Cambridge UK; ^2^ Cambridgeshire and Peterborough NHS Foundation Trust Cambridge UK; ^3^ Wolfson Brain Imaging Centre University of Cambridge Cambridge UK; ^4^ Institute of Cognitive Neuroscience UCL London UK; ^5^ Jane and Terry Semel Institute for Neuroscience and Human Behavior University of California Los Angeles California USA; ^6^ Department of Biomedical Engineering Boston University Boston Massachusetts USA; ^7^ Department of Psychiatry Warneford Hospital Oxford University Oxford UK; ^8^ Department of Brain Sciences Imperial College London London UK; ^9^ German Centre for Neurodegenerative Diseases (DZNE) Magdeburg Germany; ^10^ Inserm, Institut de Neurosciences Montpellier France; ^11^ Centre for Dementia Prevention Western General Hospital University of Edinburgh Edinburgh UK; ^12^ Sheffield Institute for Translational Neuroscience University of Sheffield Sheffield UK

**Keywords:** Alzheimer's disease, entorhinal cortex, path integration, preclinical, virtual reality

## Abstract

**INTRODUCTION:**

Entorhinal cortex (EC) is the first cortical region to exhibit neurodegeneration in Alzheimer's disease (AD), associated with EC grid cell dysfunction. Given the role of grid cells in path integration (PI)–based spatial behaviors, we predicted that PI impairment would represent the first behavioral change in adults at risk of AD.

**METHODS:**

We compared immersive virtual reality (VR) PI ability to other cognitive domains in 100 asymptomatic midlife adults stratified by hereditary and physiological AD risk factors. In some participants, behavioral data were compared to 7T magnetic resonance imaging (MRI) measures of brain structure and function.

**RESULTS:**

Midlife PI impairments predicted both hereditary and physiological AD risk, with no corresponding multi‐risk impairment in episodic memory or other spatial behaviors. Impairments associated with altered functional MRI signal in the posterior‐medial EC.

**DISCUSSION:**

Altered PI may represent the transition point from at‐risk state to disease manifestation in AD, prior to impairment in other cognitive domains.

## 1 BACKGROUND

Alzheimer's disease (AD) is the leading cause of dementia and mortality, with limited treatment options.^1^ One major reason for the historic failure of therapeutic trials is the difficulty in identifying the first onset of clinically relevant disease when interventions may have maximal value. While earlier detection of AD pathology is now possible with scalable tests for amyloid and tau biomarkers,^2^ these alone do not indicate the onset of clinical cognitive decline.^3^ Prevailing models of AD progression, which describe early damage to the entorhinal cortex (EC) and hippocampus within the medial temporal lobes (MTL), propose that cognitive changes are absent in preclinical AD.^4^ However, subtle deficits on bespoke MTL‐supported cognitive tests are found in asymptomatic people at risk of AD who appear unimpaired on standard neuropsychological tests. Increased physiological risk of AD is associated with poorer allocentric spatial memory, which is the ability to recall viewpoint‐independent topographical details of scenes.^5^ Familial AD genetic risk gene carriers are impaired on a visual short‐term associative memory binding paradigm,^6^ while carriers of the apolipoprotein E (*APOE*) ε4 allele, the main genetic risk factor for sporadic AD, consistently perform worse on tests of path integration (PI).^7^, ^8^, ^9^

PI is a behavior of high interest in early AD as it is thought to be subserved by spatially modulated grid cells in the EC,^10^ the first neocortical region to exhibit tau pathology and neurodegeneration in AD.^11^ PI represents a form of navigation in which self‐motion cues are used to estimate environmental position. In an AD mouse model, EC tau pathology was associated with grid cell dysfunction and impaired spatial behavior.^12^ Human studies have found that in patients with mild cognitive impairment (MCI) due to AD, PI error correlated with levels of cerebrospinal fluid (CSF) tau and EC volume,^13^ while in *APOE* ε4 carriers, EC grid‐like functional magnetic resonance imaging (fMRI) signal was reduced.^7^

This study therefore tested the hypothesis that EC‐related PI impairment represents the first cognitive manifestation of AD, with two predictions. First, that PI measured with immersive virtual reality (VR) is impaired in people at risk of AD, independent of risk factor type. Second, that the PI deficit would occur prior to impairment in other cognitive domains potentially affected in preclinical AD. Given that PI has not yet been assessed in preclinical AD in relation to brain structure and pathology, we additionally explored these measures using 7T ultra‐high field magnetic resonance imaging in a subset of participants.

## 2 METHODS

### 2.1 Participants

Participants were recruited from the PREVENT Dementia prospective cohort study^14^ of cognitively healthy individuals aged between 40 and 59 using newsletters and invitations during Year Two or Five study follow‐up visits across four UK sites (London, Cambridge, Oxford, and Edinburgh). Power calculations derived from our study in patients with MCI^13^ based on the primary outcome measure of our VR PI task, Euclidean location error in meters, showed that 25 matched pairs of higher and lower risk participants would have allowed us to show a mean group difference of 0.60 with an alpha risk = 0.05 and a power of 0.80. One hundred twenty‐three potential participants initially expressed interest in taking part. One potential participant was not suitable due to contraindications to using immersive VR (severe motion sickness) and 22 participants later declined, resulting in 100 people giving written informed consent for their participation. One participant was excluded from analyses due to incomplete data from the immersive VR task, leaving a final sample of 99.

**RESEARCH IN CONTEXT**

1. **Systematic review**: While several studies have shown altered spatial navigation in people at risk of Alzheimer's disease (AD), none have compared different spatial behaviors across several AD risk factors.
2. **Interpretation**: Entorhinal cortex (EC)–based path integration—but not other spatial or non‐spatial behavior—is selectively impaired in midlife people with multiple different risk factors. This is consistent with the EC being the site of initial tau deposition in AD and with pathology disrupting the spatially‐related firing of EC neurons.
3. **Future directions**: The possibility that impaired path integration may represent the transition from at‐risk status to clinical disease onset will be investigated in a follow‐on study testing presymptomatic familial AD mutation carriers. Parallel work in AD mice will investigate the relationships among tau spread, EC neuronal activity, and path integration. This cross‐species work will deliver crucial, currently missing, insights into how AD cellular pathology is linked to clinical manifestation.

We stratified participants according to three major late‐onset AD risk factors (Table 1), which we investigated both alone and in combination: (1) parental family history (FH) of dementia (*n* = 61), associated with a 3‐fold increased risk;^15^ (2) the Cardiovascular Risk Factors, Aging and Dementia Study (CAIDE) risk score derived from physiological variables including vascular health indicators, physical activity, and education, associated with greater vascular pathology, tau accumulation, and MTL atrophy over time;^16^ and (3) the *APOE* ε4 allele (*n* = 32), associated with 3‐fold increased risk.^17^ Given that females have higher dementia prevalence,^18^ show diverging pathology progression in early AD stages,^19^ and that navigational strategies differ between males and females,^20^ participants were also stratified by sex (Table S1 in supporting information).

**TABLE 1 alz13733-tbl-0001:** Demographics for all participants stratified either by family history or *APOE* ε4 status.

	Family history positive	Family history negative		*APOE* ε4 positive	*APOE* ε4 negative		Whole sample
Characteristic	*N* = 61	*N* = 38	*P*	*N* = 32	*N* = 65	*P*	*N* = 99
Sex							
Female (%)	41 (67%)	23 (61%)	*0.60* *^a^*	19 (59%)	43 (66%)	*0.70* *^a^*	64 (65%)
Age							
Mean years ± SD (range)	57.1 ± 5.16	55.8 ± 5.45	*0.20*	55.2 ± 5.46	57.2 ± 5.14	*0.08*	56.6 ± 5.28 (43 – 66)
Education							
Mean years ± SD (range)	17.0 ± 3.12	16.8 ± 2.86	*0.70*	16.8 ± 2.86	17.0 ± 2.93	*0.90* *^b^*	16.9 ± 3.01 (10 – 24)
Ethnicity							
White (%)				31 (97%)	63 (97%)	*0.70* *^a^*	
Black (%)				0	1 (2%)		
Asian (%)				1 (3%)	0		
Indian subcontinent (%)				0	1 (2%)		
*APOE* ε4							
Positive (%)	18 (30%)	14 (37%)	*0.60* *^a^*	–	–		32 (32%)
NA (%)	2 (3%)	0		–	–		2 (2%)
Family history ^†^							
Positive (%)	–	–		18 (56%)	41 (64%)	*0.60* *^a^*	61 (62%)
Family history type							
Maternal (%)	27 (44%)	–		10 (56%)	15 (37%)	*0.40* *^a^*	–
Paternal (%)	42 (69%)	–		11 (61%)	31 (76%)	*0.30* *^a^*	–
Both (%)	8 (12%)	–		3 (17%)	5 (12%)	*1.00* *^a^*	–
CAIDE DRS							
Mean score ± SD (range)	5.12 ± 2.20	4.87 ± 2.23	*0.40* *^b^*	4.94 ± 2.33	5.06 ± 2.16	*0.50* *^b^*	5.02 ± 2.21 (0 – 11)
NA (%)	3 (5%)	0		0	1 (2%)		3 (3%)

Abbreviations: *APOE*, apolipoprotein E; CAIDE, Cardiovascular Risk Factors, Aging and Dementia Study; DRS, dementia risk score; SD, standard deviation.

^a^ Pearson chi‐square test.

^b^ Wilcoxon rank sum test.

^†^ Parental.

Of these 99 participants, 55 additionally gave consent to take part in a 7 Tesla MRI scan and were similarly stratified (Table S2 in supporting information). Inclusion criteria were participation in the amyloid positron emission tomography (PET) PREVENT sub‐study or giving a CSF sample within the main PREVENT program, and an exclusion criterion was contraindication to scanning at 7T. One participant was excluded from analysis due to incomplete data from the immersive VR task, leaving a final sample of 54.

This study and the main PREVENT program were performed in accordance with the Declaration of Helsinki and each was respectively approved by institutional review boards at the National Healthy System London Camberwell St‐Giles (ref. 12/LO/1023) and West London Research Ethics Committees (ref. 18/LO/2418).

### 2.2 Risk factor characterization

Risk factor statuses were collected as part the main PREVENT Dementia program at each site.^14^, ^21^ When reporting the results, we considered family history and *APOE* ε4 status as more hereditary risk factors, and the CAIDE dementia risk score (DRS) as a more physiological‐based risk factor.

#### 2.2.1 Family history status

Participants self‐reported parental family history status with details on dementia type and age of diagnosis if applicable and known. Reported parental dementia types from participant history were 86% either AD or AD with mixed vascular pathology, 5% Parkinson's or Lewy body dementia, and the remaining 9% unknown. Participants were classified as FH+ if they had positive histories on either maternal, paternal, or both sides. For estimated years to onset of dementia calculations, if both parents were diagnosed, the parent with the earlier onset age was used.

#### 2.2.2 *APOE* ε4 genotyping

Genomic DNA was isolated from whole blood samples collected during PREVENT visits and genotyping was performed using the TaqMan polymerase chain reaction (PCR)‐based method as previously described.^22^ Participants were classed as *APOE* ε4+ if they carried either one or two allele copies.

#### 2.2.3 CAIDE DRS

The CAIDE DRS is a physiological‐based risk scoring tool derived from a prospective cohort study that identified weighted variables in midlife predictive of future dementia.^23^ It has since been validated in additional populations against CSF and neuroimaging measures, with finalized variables including age, sex, education, hypertension, cholesterol, physical activity levels, and body mass index. In this study, CAIDE score was used without *APOE* ε4 status to examine the effect of predominantly modifiable risk factors on navigation performance. It was used as a continuous variable on a scale of 0 to 15 in all analyses except for the “out of bounds” trials analysis, in which above/below median CAIDE groups were established. Statistical analysis with the CAIDE did not involve controls for age, sex, and education as they are included within the score. Cohort baseline measures were used to calculate scores.

### 2.3 Virtual reality PI task

All PI task data were collected at the University of Cambridge, the methods of which have previously been described elsewhere.^13^ In brief, the task required participants to complete a triangle by walking between three numbered[Table alz13733-tbl-0001] cones presented sequentially at eye level within an open field virtual environment viewed through a head‐mounted display. The open field was bordered by navigational features projected at infinity to represent boundary cues, with no local landmarks, to prevent use of egocentric beaconing strategies.^24^ An auditory stimulus sounded at the appearance of each cone to prompt participants toward the next cone, and cones disappeared when reached. After walking the two outward legs to reach cone three from cone one (the “outward path”), participants were instructed to return to their remembered location of cone one (the “return path”) and press a trigger on the hand‐held controller. This logged their estimated location and ended the trial.

To examine the effect of supportive environmental cue availability on PI performance, three different conditions for the return path were used. Each condition entailed a change to the environment appearance when participants reached cone three to initiate the return path: condition A, no change with all cues available; condition B, removal of surface texture; condition C, removal of distal landmarks.^13^ In addition, three different environments with varying appearance were used to maintain engagement in the task. Participants performed 12 trials per condition, totaling 36 per participant. Testing time varied from 30 to 60 minutes depending on participant walking speed and optional rest breaks. The order of conditions presented to participants was pseudo‐randomized to remove order effects and ensure participants did not become over reliant on external allothetic versus idiothetic cues to solve the task. The locations of cones and configuration and size of triangles were also pseudo‐randomized.

The task was administered with immersive VR using the HTC Vive VR hardware system and Steam VR software. Initially, this was run on the MSI VR One laptop with Intel Core i7‐7820HK, 16GB RAM, and GeForce GTX 1080, which was worn as a backpack to enable free, untethered participant movement during the task. However, equipment failure during data collection necessitated replacing the laptop with the Dell Desktop PC Precision 6820 Tower X‐Series with Intel Core i9‐10900 and GeForce RTX 2080. To enable free movement, the Vive Wireless Adaptor was additionally used. External base stations mapped out a 4 × 4m^2^ virtual test space within which participant location and task responses were tracked with a sampling rate of 0.1 second to provide raw coordinate data. Triangle return path distances ranged from 3.6 to 4 m to vary PI difficulty and at least 1 m of clear space bordered the test space. For safety precautions, a researcher was in close proximity at all times, and an “out of border” warning message appeared in participants’ line of vision to discourage walking if they moved 30 cm beyond the test space border (see Supplementary Materials in supporting information). Prior to the task start, participants had the opportunity to explore the environment in a short 20 second habituation period and complete five practice trials for which feedback on performance was given. Participants were instructed to complete the task as quickly and accurately as possible, using whatever strategy they liked, but were discouraged from retracing their outward path via cone two to estimate where cone one was. No performance feedback was given during the remaining trials.

### 2.4 Comparator neuropsychological tests

During PREVENT study visits, participants completed the digital computerized assessment of adult information processing (COGNITO) test battery^25^ and additional stand‐alone cognitive assessments to assess global and individual cognitive domain function. In this study, measures of allocentric (4 Mountains Test [4MT])^26^ and egocentric (Virtual Supermarket Trolley Task [VSTT])^27^ processing, episodic and visual association memory (Visual Short‐Term Binding Test; COGNITO Name‐Face Association and Narrative Recall),^6^ and global cognition (Addenbrooke's Cognitive Exam III)^28^ were selected from these PREVENT assessments to compare to PI, for which the administrative procedures and task details have previously been described.

### 2.5 MRI acquisition

MRI data were acquired on a 7T Terra MR system (Siemens) with a Nova Medical 1Tx/32Rx head coil at the Wolfson Brain Imaging Centre, University of Cambridge. Sequences were aligned to the 7T UK harmonization protocol.^29^ First, a whole brain T1‐weighted magnetization prepared 2 rapid acquisition gradient echo (MP2RAGE) volume was obtained (repetition time [TR] 3500 ms, echo time [TE] 2.58 ms, inversion time [TI] 1 = 725 ms, TI 2 = 2159 ms, resolution 0.7 mm isotropic, generalized autocalibrating partial parallel acquisition [GRAPPA] = 3, matrix size = 224 × 224 × 157, flip angle [FA] 1 = 5°, FA 2 = 2°). Second, a high‐resolution partial T2‐weighted structural volume was obtained with slices orientated perpendicular to hippocampal long axis (TR 8080 ms, TE 76 ms, resolution 0.4 mm x 0.4 mm, slice thickness 1 mm + 10% gap, GRAPPA = 2, matrix size = 224 × 224 × 54, FA = 60°). Finally, the fMRI session was run using T2*‐weighted gradient echo planar images (EPIs) with an in‐plane resolution of 1.5 mm x 1.5 mm (42 axial slices, TR 2531 ms, TE 22 ms, slice thickness 1 mm, GRAPPA = 2, FA = 73°, matrix size = 192 × 192 × 42). Slices were centered on and orientated parallel to the hippocampus long axis. Higher between‐plane resolution aimed to minimize dropout due to partial volume effects, while lower within‐plane resolution aimed to increase signal‐to‐noise ratio. Total scan time was 75 minutes.

### 2.6 MRI pre‐processing and analysis

T1‐weighted structural images were produced via offline PSIR (phase‐sensitive inversion recovery) reconstruction on all MP2RAGE data. T1‐weighted and T2‐weighted images were manually inspected for artifacts and bias field corrected using the N4 algorithm.^30^ The EPI series were preprocessed using FSL v5.0.8 (FMRIB) and SPM12 (http://www.fil.ion.ucl.ac.uk/spm/) in MATLAB 2019a (MathWorks). First, images were manually inspected for artifacts and corrected for distortions using a reverse phase encoded method via FSL‐topup.^31^ EPIs then underwent motion correction with SPM realign, reslice, and slice time correction to account for differences in interleaved slice acquisition times. All analyses were carried out on native space images to prevent potential signal distortions during non‐linear normalization to a common space. All images (EPIs T1‐weighted and T2‐weighted) were co‐registered using the default Advanced Normalization Tools (ANTs) linear transformations.^32^ In cases of registration failure, images were manually co‐registered in ITK‐SNAP.^33^

Regions of interest (ROIs) were selected a priori either due to their hypothesized role in navigation functions or early susceptibility to pathology in initial AD stages. These included the whole and posterior‐medial EC, hippocampal subfields (subiculum and CA1), retrosplenial cortex, and posterior–cingulate cortex. For non‐medial temporal lobe ROIs, T1‐weighted images underwent normalization to MNI305 atlas space, brain extraction, tissue segmentation (CSF, gray matter, white matter), and parcellation according to the Desikan–Killiany atlas using the FreeSurfer image analysis suite (v7.1.0, https://surfer.nmr.mgh.harvard.edu/). Pial surface misplacements and erroneous white matter segmentation were manually corrected on a slice‐by‐slice basis if individual brain processing failed. The isthmus cingulate ROI was used as a proxy retrosplenial cortex ROI mask following previous work.^13^

Medial‐temporal ROIs were created in the subject's T2‐weighted space using a semi‐automatic approach with the Automatic Segmentation of Hippocampal Subfields (ASHS) software V2.0^34^ and IKND Magdeburg 7T multi‐template atlas.^35^ ASHS outputs per hemisphere per participant were manually inspected and corrected. This included erroneously included CSF and meninges voxels or misplaced gray/white matter or anterior/posterior borders judged using established heuristic rules.^34^, ^35^ Specifically, for the posterior–medial EC, which is not available in ASHS, a publicly available common‐space mask based on diffusion tensor imaging connectivity^36^ was used to manually trace posterior–medial EC voxels visible on the high‐resolution T2 using ITK‐SNAP.^33^ The mask was warped into individual native space via (1) average group T1‐weighted template made using the opensource toolkit ANTs (v2.3.4) diffeomorphic template construction algorithm^37^ and (2) individual participant T1‐ and T2‐weighted images, all co‐registered and transformed using ANTs.^32^ In some participants the warped common space posterior–medial entorhinal mask extended anteriorly beyond the field of view of the T2‐weighted image; these voxels were not included in the final ROI mask. Manual segmentation was performed by two raters with an average Dice similarity coefficient across all ROIs for five subjects of 0.92, indicating good inter‐rater reliability.

### 2.7 fMRI grid cell task

The grid cell functional task was presented as three blocks of a 10 minute video followed by a short memory test. The video required participants to watch themselves be passively navigated through a virtual room from a first‐person perspective and learn the locations of seven target objects in the room. Target objects were everyday, household items and were highlighted by a hovering orange cone above them. Objects appeared progressively as movement within the video proceeded to cover the entire space. Movements were sequences of forward translations and rotations of varying angles. Passive participant viewing without movement control aimed to reduce motion induced through use of a joystick or button box and control the degree of room exploration per participant. The virtual room consisted of four gray walls decorated with different items to provide orientation cues, which stayed the same across all three videos—only the target objects within the room changed. When the video ended, participants were shown three images per each object from the room; one in the correct object location and two distractor images. Participants were required to select the correct image via a corresponding button on the button box, with accuracy and reaction time recorded. The next block began after the set of object location questions ended. The grid cell task was programmed in Unity software (V2018.2.9f1) and was presented on an MRI‐compatible LCD screen that participants viewed through a mirror mounted on the head coil at an angle of 14°. Before scanning, participants were given verbal instructions and shown a 3 minute practice video of the task. Participant position and heading directions were sampled roughly every 20 ms during the video, which enabled us to approximate grid event timestamps, with all participants viewing the same video per block.

### 2.8 Statistical analyses

Statistics were performed in R v4.0.4 using the lme4^38^ and emmeans^39^ packages. Differences between participant demographics based on risk factor and sex stratification were assessed via *t* tests or non‐parametric Mann–Whitney *U* test for continuous variables (depending on the normality of the data), and chi‐square test for categorical variables. Where appropriate in the following sections, all model residuals were inspected for deviations from homoscedasticity and normality.

#### 2.8.1 Virtual reality PI task

All outcome measures and variables were extracted and calculated in MATLAB 2019a. Each trial was manually inspected for data integrity. Trials in which participants adopted a “retracing” strategy or did not initiate the return path were excluded (0.6% of total trials). Trials in which participants went beyond the virtual test space boundary (“out of bounds”) were also excluded (34.6% of total trials), in line with previous work.^13^ These trials were qualitatively different to normal trials, as participants received an extra spatial cue informing their current position when the boundary was reached. We used chi‐square tests to assess if proportions of out‐of‐bounds trials differed between stratified groups, and a variety of control analyses to assess whether the exclusion of trials impacted the main findings (see Supplementary Materials).

Following previous research,^40^, ^41^ the primary outcome measure for the PI task was Location Error in virtual meters, reflecting the Euclidean distance between estimated and actual locations of cone one. We calculated distances using Equation (1), with coordinates of cone one estimated (*X*_1_,*Y*_1_) and true (*X*_2_,*Y*_2_) locations for Location Error:

(1) $$Distance\;=\;\sqrt{{\left(X_{1}-X_{2}\right)}^{2}+\;{\left(Y_{1}-Y_{2}\right)}^{2}}$$

First, an interaction effect of all risk factors, return condition type, and sex on location error was assessed via a mixed linear model in accordance with our primary research question. We chose mixed modeling given the clustered and incomplete nature of the data (12 trials per one of three return conditions, with out‐of‐bounds trials excluded), in line with previous PI literature.^8^, ^13^ Covariates included age, years of education, and a random intercept of (1) trial order number and (2) unique participant identifier with random slopes of return condition type to assess for participant variance across repeated trials.

In a second analysis we used multiple linear regression models to explore the change in average performance across conditions based on interactions of condition types in the mixed model results. We separately predicted change in location error between baseline and no optic flow conditions and between baseline and no distal cues conditions; in each case mean baseline performance was subtracted from the other conditions per participant to derive the outcome measure. An interaction effect of all risk factors and sex on this change in mean location error was examined in accordance with our primary research question. Covariates included age and years of education. Interaction effects were tested with analyses of variance (ANOVA) tests and post hoc contrasted pairwise using *t* tests Tukey‐corrected for multiple comparisons.

Two additional outcome measures of Absolute Angular and Distance Error decomposed the Location Error into linear and rotational error contributions. Distance error reflects the accuracy of participant distance estimation of the return path length and was determined using the absolute values of *D_true_* – *D_estimated_*, where *D_true_* refers to the true distance between cone three and cone one, and *D_estimated_* the participants’ estimated distance between cone three and their triggered position. Angular error reflects the accuracy of participant rotation at cone three to return to cone one and was determined using the absolute values of *A_true_* – *A_estimated_*, where *A_true_* refers to the true rotation angle from cone three and cone one, and *A_estimated_* the participants’ estimated angle between cone three and their triggered position. Each angle (θ) was calculated using Equation (2):

(2) $$\theta =atan2d\left(\vec{v1}\;\times \;\vec{v2},\;\vec{v1}\;.\;\vec{v2}\right)$$

where $\vec{v1}$ represents the trajectory vector between cones two and three, $\vec{v2}$ the vector between cone three and either the true or estimated location of cone one. atan2d is a MATLAB function that takes the arctangent (in degrees) of the cross and dot product of two vectors to derive the angle between them. Another series of mixed linear models with the same covariates and predictors were used to assess the effect of risk factors and sex on change in absolute angular and distance error.

We created a signed allocentric angular error outcome measure to assess the directionality of angular errors. The angular difference was mapped in the interval $\left[-180^{\circ },\;180^{\circ }\right]$ using the MATLAB wrapto180 function as $wrapTo180(A_{true}-A_{estimated})$. Therefore, negative values indicate overturning in the allocentric point of view, whereas positive values indicate underturning in the allocentric point of view. For instance, if $A_{true}=120^{\circ }$ and $A_{estimated}=-220^{\circ }$, then the signed allocentric angular error will be $-20^{\circ }$ (an overturning of $20^{\circ }$), whereas the absolute angular error is $340^{\circ }$. We additionally calculated this for out‐of‐bounds trials by taking the position of the boundary collision as a proxy measure of initial angular estimate for the return path. This enabled us to circumvent the data bias of overturning errors introduced by excluding out‐of‐bounds trials (see Supplementary Materials).

Finally, we used ANOVA nested model comparison to explore the proportion of variance explained by different risk factors. We repeated the above linear regression models on change in performance from baseline to “no distal cues” using individual risk factors interacting with sex, controlled for age and education. We conducted *F* tests to compare adjusted *R* ^2^ between each of these models against the full model used above.

#### 2.8.2 Comparator neuropsychological tests

We used multiple linear regression models to explore interactive effects of all risk factors and sex on task performance for the comparator assessments, in keeping with the PI analysis. We additionally ran separate linear regression models per individual risk factor to explore univariate effects. Covariates always included age, years of education, and PREVENT visit date to confirm that differences in time‐locking across visit dates for PREVENT cognitive assessments and the PI study participation did not affect results. To compare the relative predictive value of PI to other assessments, we performed cross‐validated, logistic regression using elastic‐net regularization to optimize the area under the curve (AUC) of the receiver operating characteristic (ROC). We predicted a “double‐risk” status (FH+/*APOE* ε4+ vs. any other combination, based on earlier model performance) using performance on the VR PI task (viz. change in location error from baseline to “no distal cues” condition) plus performance on the other tasks explored above, as well as age, sex, and education. We used 1000 random permutations of training versus test.

#### 2.8.3 ROI structure

For the MTL ROIs, regional T2‐weighted volumes were extracted using the Insight Toolkit Convert3D software (www.itksnap.org/c3d). T1‐weighted isthmus cingulate and posterior cingulate regional volumes, as well as T1‐weighted total intracranial volume, were extracted using FreeSurfer v7.1.0 (as described above). To reduce multiple comparisons, volumes from the left and right hemisphere were summed, and to correct for variations in brain size, volumes were expressed as a percentage of total intracranial volume to estimate relative gray matter. We ran an additional analysis using raw volumes with total intracranial volume as a covariate to confirm this analysis approach did not alter results.

First, we used multiple linear regressions predicting each individual ROI per each individual risk factor (FH, *APOE* ε4, CAIDE) to assess effects of risk on brain structure. We then used a three‐way interaction between each ROI volume, risk factor status, and sex to predict change in location error across conditions to assess effects of risk on brain–behavior relationships established in earlier analyses. All models had covariates of age and years in education. The false discovery rate method was used to correct for planned multiple ROI comparisons per each risk factor and response variable.^42^

Finally, to establish if multivariate contributions of all ROIs better explained structural brain–behavior relationships, we used a multiple regression with all ROIs as predictors with age, sex, and education covariates.

#### 2.8.4 fMRI grid‐cell‐like representations

Putative measures of grid cell codes were extracted from the pre‐processed EPIs using a reproducible, standardized approach with the GridCAT v1.04^43^ and CircStat^44^ toolboxes in MATLAB (2017b, MathWorks) and SPM12 (http://www.fil.ion.ucl.ac.uk/spm/). The spatial properties of the grid patterns and the non‐linearity of the mapping from location through neural activity to the macroscopic blood oxygen level–dependent (BOLD) fMRI signal suggest that grid population activity could be detectable in fMRI. Namely, the orientations of the grid‐firing patterns of both local and distant grid cells are clustered, despite differences in grid scale.^45^, ^46^ Thus, the different neural firing dynamics when running in directions aligned versus misaligned to grid axes (i.e., some cells firing a lot, others a little, vs. all cells firing an intermediate amount) would generate different fMRI signal strengths.^47^

Data analysis here used two general linear models with parametric modulators of *sin* and *cos* to (1) estimate individuals’ grid orientation (in 60° space) using the time‐varying translation events in half of the data, and (2) derive measures of model fit using this estimated orientation for modeling translation events in the remaining data. Data were partitioned using odd/even translation events and nuisance regressors for head movement were included in the general linear models. Only voxels masked by the right posterior–medial entorhinal subdivision mask were used to calculate outcome metrics, in line with previous findings.^7^, ^8^, ^47^ The primary outcome measure was grid cell–like representation magnitude, in which a higher magnitude entailed better fit of the general linear model for the estimated mean grid angle.^43^ Secondary measures of between‐voxel orientation coherence and within‐voxel orientation coherence over time, respectively, provided measures of spatial and temporal stability. Spatial stability was calculated using Rayleigh test for non‐uniformity of circular data using voxelwise mean grid orientations. Temporal stability was calculated by comparing orientation values within voxels between the first and second half of each scanning run, expressed as the percentage of voxels with a change of less than 15° in orientation. We included these secondary measures because temporal but not spatial stability was demonstrated to be the cause of low‐magnitude grid codes in young *APOE* ε4 carriers relative to non‐carriers.^7^

The fMRI grid outcome metrics were normally distributed, so one‐sample *t* tests were used to assess if magnitudes and spatial stabilities of grid cell–like representations were significantly different from zero and if temporal stability was significantly different from chance (50%) across all participants. Differences in metrics between FH/*APOE* ε4 risk groups and sex were compared using Welch two‐sample *t* tests given unequal variances while associations between metrics and age or CAIDE lifestyle risk score were calculated with Pearson correlations.

We used multiple linear regression models to test if (1) metrics predicted change in location error from baseline to no distal cues conditions and following on from this (2) if a three‐way interaction between grid cell–like representation magnitude, risk status, and sex modulated this relationship. We always used covariates of age and education, and sex additionally in the first regression models. To assess the continuous × continuous CAIDE interaction with grid‐activity magnitude, we used Johnson–Neyman intervals to explore the range of CAIDE values for which the association between grid activity and PI performance was significant.

We additionally performed a range of control analyses (see Supplementary Materials). Grid cell–like representation magnitudes were calculated using either four‐fold, five‐fold, or seven‐fold rotational symmetry (instead of expected six‐fold) to confirm the specificity of the grid‐characteristic patterns, and a one‐fold (or unimodal) grid pattern in an exploratory analysis. Temporal signal‐to‐noise ratio for the posterior–medial EC was calculated by dividing voxel‐wise mean time series by its standard deviation, and this was associated against grid cell–like representation magnitudes using Pearson correlation.

## 3 RESULTS

### 3.1 PI impairments across risk factors

**FIGURE 1:**
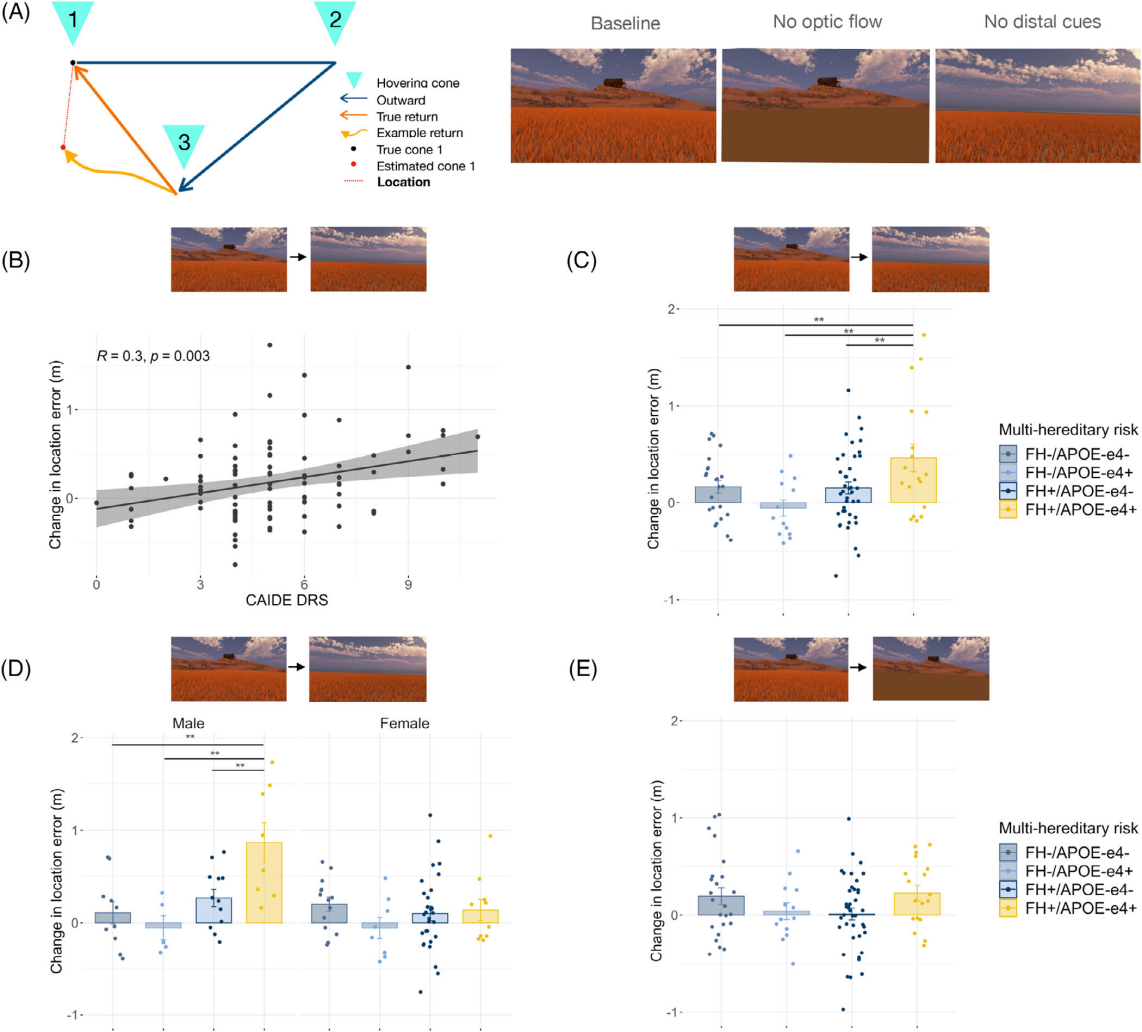
Hereditary and physiological risk factor PI impairments. A, The PI task schematic and three return condition types. B, Higher CAIDE dementia risk score significantly correlated with decline in performance (viz. increase in location error) on no distal cues condition relative to baseline in both sexes. C, Decline in performance on no distal cues relative to baseline was largest in FH+/*APOE* ε4+. D, FH+ and *APOE* ε4+ performance decline on no distal cues relative to baseline was specific to males (combined for display). E, FH+/*APOE* ε4+ or CAIDE (not pictured) decline in performance on no optic flow condition relative to baseline was not significant. ** *P*_Tukey_ < 0.01. *APOE*, apolipoprotein E; CAIDE, Cardiovascular Risk Factors, Aging and Dementia Study; FH, family history; PI, path integration

While we found no overall main effects of FH, *APOE* ε4, or CAIDE score on location error when modeling individual trials with a mixed model approach (all *F *≤ 1.17, *P *≥ 0.283), several significant interactions among risk factors, return condition, and sex were present (all *F *≥ 2.90, *P *≤ 0.050). To study these further, we examined performance on each of the two manipulated return conditions relative to baseline (which additionally controls for individual differences in overall performance; Figure 1A) using linear regression to assess the interactive effects of all risk factors and sex together. We found significant worsening of PI performance after removal of the distal orientation cues (return condition three) across all individuals with elevated AD risk; namely, both a main effect of CAIDE (*F*_1,77_ = 11.04, *P *= 0.001; Pearson's *r* = 0.30, *P *= 0.003; Figure 1B) and two‐way interaction effect of family history and *APOE* ε4 status (*F*_1,77_ = 8.43, *P *= 0.005; hereditary x physiological interactions all *P* > 0.303). There were no effects of age or education (both *F* < 1.32, *P* > 0.254; see Table S3 in supporting information for FH/*APOE* ε4 demographic breakdown). Post hoc analyses on the two‐way interaction showed that worsening performance was greatest in individuals with both FH+ and *APOE* ε4+ (all t ≥ 3.28, *P_Tukey_*_ _≤ 0.008; Figure 1C).

We additionally found that both FH and *APOE* ε4 also interacted with sex (both *F* ≥ 8.11, *P* ≤ 0.006), with detrimental risk effects specifically occurring in males (Figure 1D). We further explored the male‐specific FH+ effect using estimated years to onset of dementia (EYOD), a temporal marker of preclinical state based on the difference between participant age and their parental age of dementia onset. In this cohort with a mean EYOD of 19.3 years, lower EYOD correlated with greater location error on the no distal cues condition (Pearson EYOD *r *= 0.55, *P *= 0.010; age *r *= 0.16, *P *= 0.300).

We confirmed these combined risk factor and sex‐specific results by comparing the explained variance to simpler models (Table S4 in supporting information). The full model explained significantly more variance than the same model without a sex interaction (*F*_77,84_ = 3.05, *P *= 0.007; adjusted *R* ^2^ = 0.26). It also explained more variance than separate models with individual risk factors (all *F*_77,90_ ≥ 2.84, *P *≤ 0.002; all individual models adjusted *R* ^2^ < 0.06), but not more than the same model with CAIDE omitted (*F*_77,85 _= 1.85, *P* = 0.081; adjusted *R* ^2^ = 0.20). This may suggest that the relationship between distal cue–dependent PI performance and AD risk was predominantly driven by hereditary‐related risk factors of family history and *APOE* ε4 status, rather than the more physiological‐related CAIDE score.

#### 3.1.1 Decomposing PI impairments

**FIGURE 2:**
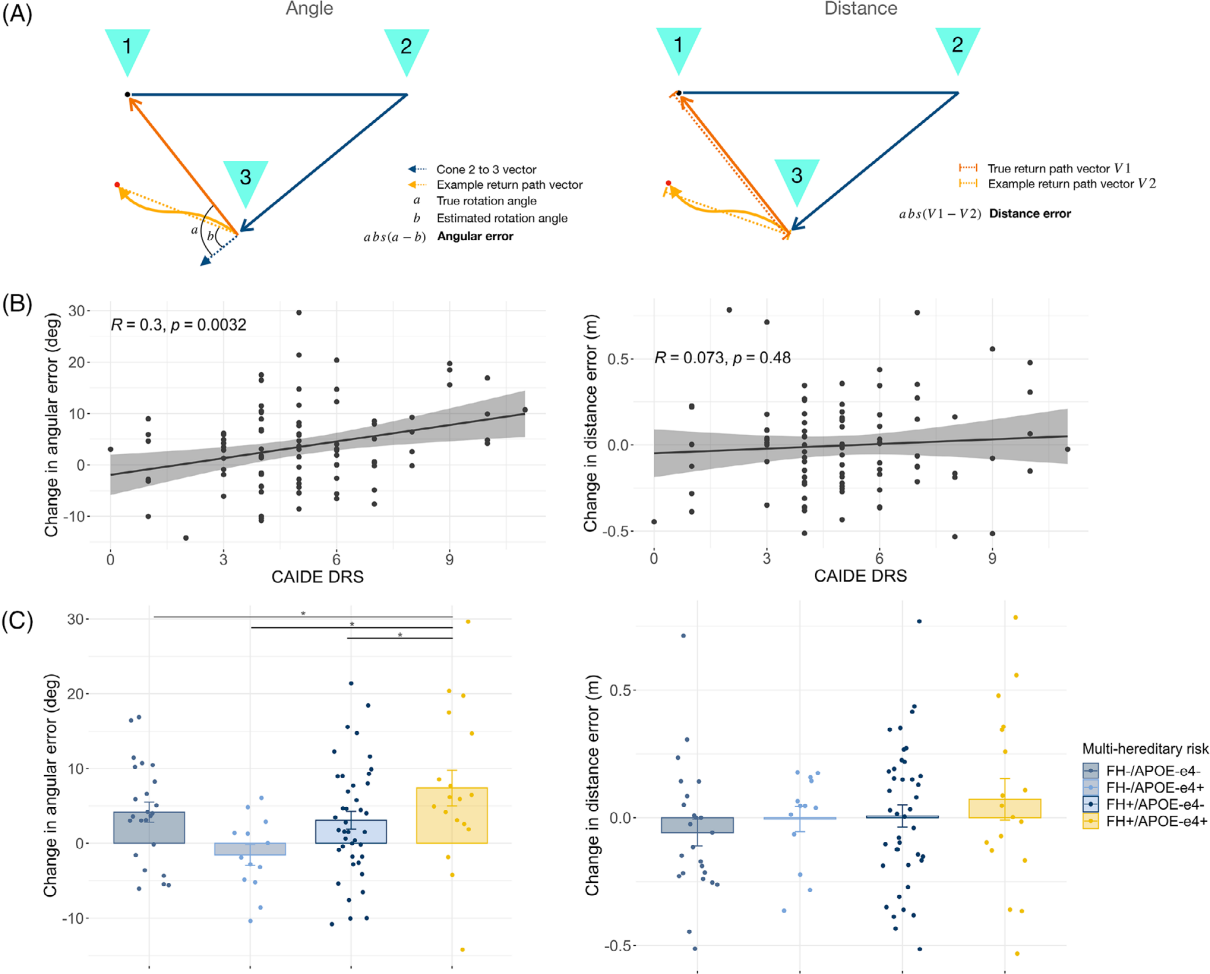
Angular rather than distance errors contributed to risk factor–associated impairment of PI after removal of distal orientation cues. A, Schematic of angular error (left) and distance error (right) calculations. B, CAIDE dementia risk score correlated with change in angular but not distance error from baseline to no distal cues. C, Multi‐hereditary risk interacted with sex for angular but not distance error changes in PI. *P_Tukey_ < 0.05. *APOE*, apolipoprotein E; CAIDE, Cardiovascular Risk Factors, Aging and Dementia Study; DRS, dementia risk score; FH, family history; PI, path integration

In contrast to the third “no distal cues” condition, performance differences between baseline and “no optic flow” conditions (return condition two) were borderline significant for hereditary but not physiological risk factors (two‐way FH x *APOE* ε4 *F*_1,77_ = 4.42, *P *= 0.039; post hoc pairwise tests all *P*_Tukey _> 0.138; Figure 1E), indicating that the PI impairment observed across all AD risk groups related specifically to orientation cue removal. To understand this impairment further, we decomposed location error into absolute distance and angular error (Figure 2A). Using the same risk factor × sex model, we found that orientation‐related location errors in the at‐risk individuals were driven by angular not distance errors, with the same main effect of CAIDE (angular error *F*_1,77_ = 10.42, *P *= 0.002; distance error *P *= 0.500; see Figure 2B for correlations) and two‐way interactions of FH and *APOE* ε4 together or individually with sex (angular error all *F*_ _> 4.88, *P *< 0.030; distance error all *P *> 0.360; Figure 2C). By wrapping the return angles of each trial to [−180,180] allocentric space, we determined that angular errors resulted from over‐ rather than underturning (Figure S1; see Figure S2‐S3 in supporting information for control analyses).

#### 3.1.2 Performance on comparator spatial and nonspatial tests

For comparison, we tested other cognitive domains affected in preclinical AD (Table 2). Episodic memory, historically considered the domain first affected in AD, was assessed for non‐verbal name‐face associative memory,^25^ visual short‐term memory binding,^6^ and verbal narrative recall. Global cognition was indexed with the Addenbrooke's Cognitive Exam III.^28^ Other aspects of spatial behavior were tested with the VSTT of egocentric spatial orientation,^27^ reflecting medial parietal lobe function and pertinent given early amyloid beta (Aβ) deposition in this region, and the hippocampus‐dependent 4MT of allocentric spatial memory.^26^

**TABLE 2:** Summary of findings: Path integration versus other cognitive domain performance association with midlife dementia risk.

Exam	Metrics used	Relevant cognitive domain	Multivariate risk factor performance associations
Immersive virtual reality path integration task	Location error (m)	Entorhinal path integration navigation	FH, *APOE* ε4, CAIDE
Addenbrookes Cognitive Exam III (ACE)	Total score/100	Global muti‐domain cognitive function	–
Visual short‐term binding test (VSTBT)	A’ of shape‐color binding performance	Non‐verbal frontal and temporal associative memory	–
Four Mountains Task (4MT)	Total score/15	Hippocampal allocentric spatial memory	FH
Virtual Supermarket Trolley Task (VSTT)	Total orientation score/20	Retrosplenial egocentric spatial memory	–
COGNITO Name–face association	Total score/9	Non‐verbal medial–temporal paired associative memory	CAIDE
COGNITO Narrative recall	Total score/27	Verbal medial–temporal episodic memory	–

Abbreviations: *APOE*, apolipoprotein E; CAIDE, Cardiovascular Risk Factors, Aging and Dementia Study; FH, family history; PI, path integration .

Unlike PI, we found no combined interactive effects of FH, *APOE* ε4, or CAIDE on the performance of any individual comparator task (all *P *≥ 0.14; Table S5 in supporting information), but some individual risk factor effects were present. For episodic memory, no individuals at increased risk, regardless of risk factor, exhibited impairments on narrative recall or visual short‐term binding (all *P *≥ 0.100) while name–face association selectively correlated with CAIDE score (*F*_1,79 _= 21.28, *P* < 0.001; Pearson *r* = 0.41, *P* < 0.001). For spatial tests, only family history status had an effect, with FH+ individuals performing worse on the 4MT (*F*_1,74 _= 9.33, *P* = 0.003). Finally, when risk factors were separately modeled in individual risk factor x sex interaction univariate models, female FH+ performed worse than FH– on the VSTT (*t*_92_ = 3.12, *P*_Tukey_ = 0.013; two‐way interaction FH x sex *F*_1,92_ = 7.42, *P *= 0.008).

We next compared the ability of the PI task to predict “double‐risk” status (FH+/*APOE* ε4+ vs. any other combination) to that of the other cognitive tests via cross‐validated logistic regression using elastic‐net regularization to optimize the AUC of the ROC. Though the mean AUC across 1000 iterations was only 0.67, 93% of iterations included a non‐zero contribution from PI performance, whereas < 1% of iterations included a non‐zero contribution from any other cognitive test. When age, sex, or education were used as predictors, the mean AUC was 0.56. In summary, PI was the only behavior predictive of multifactor hereditary AD risk.

#### 3.1.3 Structural MRI correlates of PI impairments

In a subset of 54 participants (Table S2), ultra‐high field 7T MRI was used to assess the volume of brain ROIs associated with both navigation and early AD, namely the EC–hippocampal subfields, retrosplenial cortex, and posterior cingulate gyrus.^48^ There were no significant differences in regional volumes between high‐ and low‐risk participants across individual or interacting risk factors after multiple comparison correction (all *P*_FDR _> 0.183; Table S6 in supporting information). When predicting change in performance from baseline to no distal cues, after adjusting for age, education, and sex, there was no effect of any ROI independently, or interaction between ROI and risk factors (all *P*_FDR_ > 0.360), or even a combined effect when adding all ROIs into a single linear model (*F*_14,37 _= 1.39, *P* = 0.208).

#### 3.1.4 Functional MRI correlates of PI impairments

**FIGURE 3:**
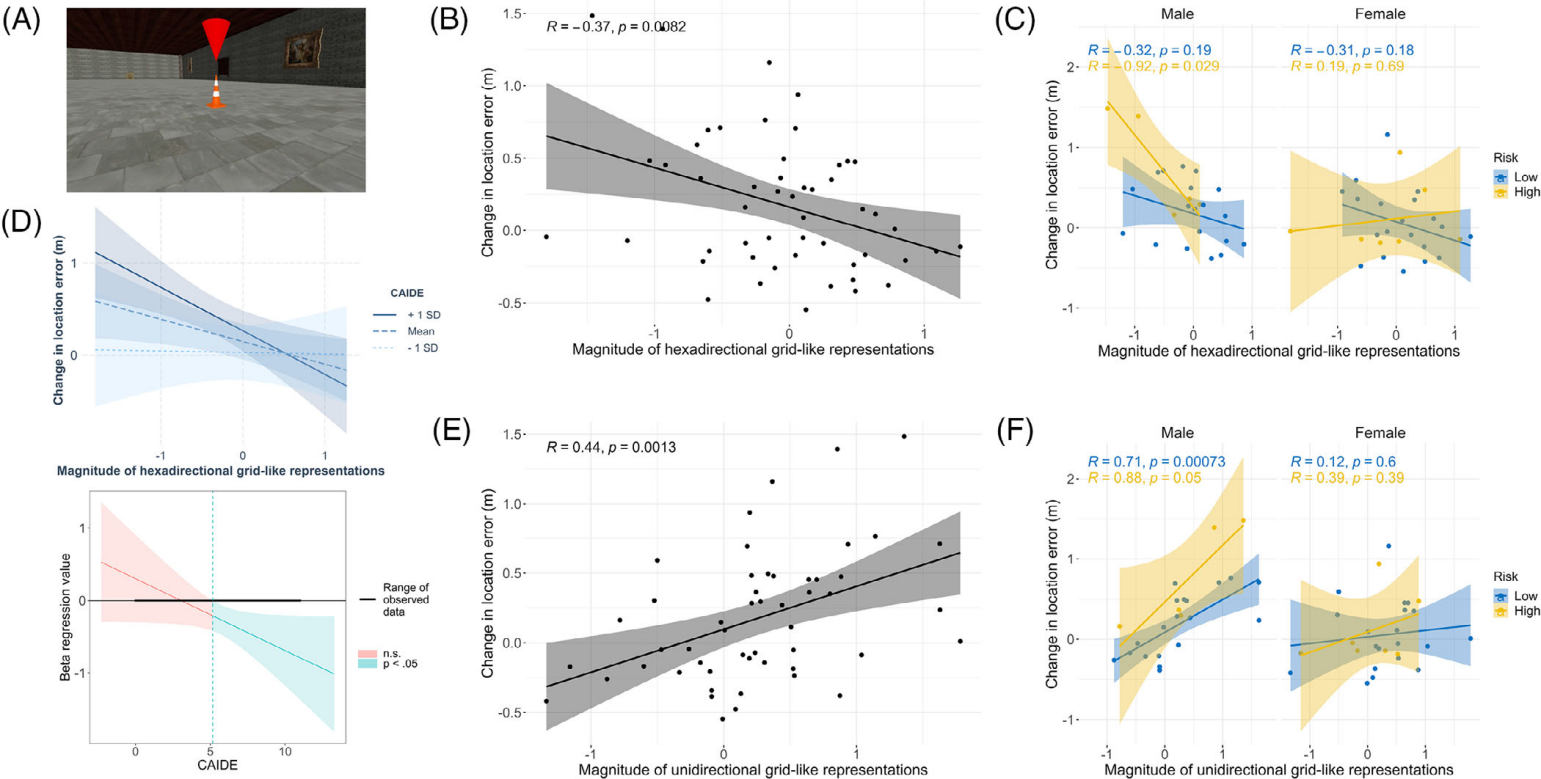
Right posterior–medial entorhinal fMRI correlates of decline in path integration performance. A, Image of object memory location fMRI task. B, Greater change in location error from baseline to “no distal cues” return condition associated with lower 6‐fold grid‐like activity, which reflects behavioral findings of showing a stronger effect in males with hereditary risk (C) or individuals with a higher CAIDE score (D). High risk is defined as FH+/*APOE* ε4+ and low risk as individual or no FH/*APOE* ε4 risk factors. E, The negative 6‐fold grid magnitudes in (B,C) might relate to a unidirectional head direction‐like signal, which was stronger in individuals that showed worse PI in the absence of orientation cues. Again, this appeared specific to males with hereditary risk (F) in an exploratory analysis. *APOE*, apolipoprotein E; CAIDE, Cardiovascular Risk Factors, Aging and Dementia Study; FH, family history; fMRI, functional magnetic resonance imaging; PI, path integration

We used a spatial memory paradigm previously found to elicit grid‐like fMRI signals in the EC^49^ (Figure 3A). We focused on the right posteromedial EC, the human homologue of rodent medial EC where most grid cells are resident and where fMRI signal was reduced in *APOE* ε4 carriers^7^ and healthy older versus younger adults.^49^ Adopting a similar analysis procedure with partitioning by odd/even grid events,^43^ we found that hexadirectional grid‐like fMRI signals were not significant in the population overall (one‐way *t* test *t*_52 _= 0.50, *P *= 0.600), but individuals with greater signals showed smaller PI performance declines from baseline to no distal cue conditions (β = −0.27 ± 0.10, *t_46_* = 2.66, *P *= 0.011; Figure 3B). Risk factors alone had no effect on the signal magnitude (all main and interaction effects *P* > 0.131). However, PI performance was predicted by a two‐way interaction between the continuous CAIDE score and grid‐like signal (*F*_1,45_ = 4.20, *P *= 0.046; Johnson–Neyman interval significant for CAIDE > 7 β = −0.47 ± 0.15, *t_45_* = 3.17, *P *< 0.001; no hereditary x CAIDE risk interaction; Figure 3D) and by three‐way interactions among individual hereditary risk factors, sex, and grid‐like signal (FH interaction *F*_1,41_ = 4.16, *P *= 0.048; *APOE* ε4 interaction *F*_1,41_ = 3.52, *P *= 0.068; Figure 3C). Including grid‐like fMRI signal as a predictor in these models provided a better fit for the PI behavioral data than null models without fMRI inclusion (all *F* > 3.42, *P* < 0.017; for controls see Supplementary Materials), suggesting that impaired PI performance across risk groups was associated with altered grid‐like fMRI signal in the posteromedial EC. More specifically, the higher risk individuals with poorer PI performance showed negative hexadirectional grid‐like activity magnitudes. Negative magnitudes related to grid‐like signal drift over time have been reported in healthy individuals and *APOE* ε4 carriers with poorer PI ability.^7^, ^8^, ^49^ However, negative grid magnitudes due to temporal signal drift were not consistent with our data partitioning procedure, which used interleaved odd/even grid events to create estimation and test data sets. We hypothesized that non–6‐fold symmetries might have contributed to the negative grid magnitudes after observing unbalanced directional sampling between partitioned sets, which we explored in a supplementary analysis. While there were no effects of 4‐, 5‐, or 7‐fold symmetries on PI performance (all *P* > 0.270), we found that a unidirectional signal consistent with head direction–like processing predicted poorer PI performance after removal of distal orientation cues (β = 0.31 ± 0.09, *t_46_* = 3.50, *P *= 0.001; Figure 3E), complementing in reverse the sex and AD risk effects observed with the hexadirectional grid‐like activity (Figure 3C and F). Across male participants only, this unidirectional signal significantly clustered around a mean direction of 181° (Figure S4 in supporting information; Supplementary Materials).

## 4 DISCUSSION

Determination of initial cognitive changes in asymptomatic individuals at risk of developing AD is a key aspect of identifying the clinical onset of AD. The importance of this identification is amplified by the advent of drug therapies with disease‐modifying potential, given the increasing evidence that such interventions may be most efficacious if applied in the earliest stages of disease. The entorhinal cortex (EC) is involved from the initial stages of AD, and that EC grid cells play a crucial role in path integration (PI), we tested the hypothesis that PI was affected in people at risk of AD prior to the onset of symptoms and prior to impairment in other cognitive domains. Consistent with this hypothesis, we found that asymptomatic individuals at risk of AD, due to either hereditary or physiological risk factors, were selectively impaired on a test of PI. Crucially, we did not find a similar impairment in other aspects of spatial behavior (allocentric spatial memory or egocentric spatial orientation) or in tests of episodic memory, including a test of visual short‐term memory binding found previously to be impaired in people with presymptomatic familial AD.

Two other observations underscore the significance of these data. First, the cohort studied were aged between 43 and 66 years and were approximately two decades younger than their estimated age of onset of dementia, indicating that this navigational impairment significantly predates clinical diagnosis. Second, impaired PI was observed in at‐risk individuals across a variety of different risk factors for AD, indicating that the effect is not specific to any individual risk factor—such as *APOE* ε4—or the underlying physiological mechanism causing the specific risk, but is a general effect, in turn raising the possibility that impaired PI may represent the inflection point in the AD trajectory from at‐risk status to disease onset.

The demonstration of an early deficit on a behavioral task based on EC function is consistent with neuropathological studies showing that the EC is the first neocortical site to exhibit neurodegeneration in AD^50^ and with animal studies showing that AD pathology in the EC is associated with disruption of neuronal activity and spatial memory.^12^ The importance of the EC in the AD pathological cascade has recently been underscored by the publication of a case report^51^ describing a member of the world's largest known kindred with autosomal dominant AD due to the *PSEN1‐E280A* mutation, who was additionally heterozygous for a rare mutation in the *RELN* gene encoding reelin and whose age of dementia onset was delayed by almost three decades compared to other kindred members. PET imaging revealed widespread Aβ and tau deposition but limited tau tangles in the EC. This relative preservation of the EC in an individual resilient to familial AD, allied to the observation that layer II EC projection neurons express reelin signaling within tau phosphorylation regulation pathways,^52^ raises new questions about the importance of studying behavioral readouts of EC function not just for early AD detection but also for the development of future therapeutic interventions.^50^

The specific PI deficit after removal of orientation cues when only self‐motion cues are available is consistent with grid cell stability being dependent on environmental boundaries.^53^ This imitates PI deficits in *APOE* ε4 carriers and may reflect difficulty in grid anchoring or an inability to use a “purer” PI strategy.^7^, ^8^ This is consistent with previous work demonstrating the importance of natural locomotion for more accurate judgments of direction in humans and more robust spatial cell activity in rodents.^54^, ^55^, ^56^ Additionally, our observation that defective angular estimation drove PI errors is also in line with increasing evidence that spatial navigation is underpinned by neuronal vector‐based coding.^57^, ^58^ Furthermore, we uncovered a sex effect, with hereditary at‐risk males preferentially impaired on PI, and a tentative univariate observation of a FH+ female impairment on the egocentric VSTT task. This may reflect sex differences in navigational strategy, with females tending toward landmark or route navigation, and males survey‐based allocentric mapping^20^ but may also reflect sex differences in AD pathological spatiotemporal progression, with greater early parietal tau pathology in females.^19^

Given that grid cell functioning is dependent on head direction, vestibular and optic flow information relayed to the EC via afferents from various brain regions including those affected in early AD, such as the medial temporal and parietal lobes,^48^ the PI impairment observed across hereditary and physiological risk factors for AD might reflect the unique vulnerability of the entire grid cell/PI network to disparate converging pathophysiological processes. These encompass tau deposition in the MTL, Aβ in the medial parietal lobe, and vascular pathology, all of which are associated with family history of dementia, *APOE* ε4, and CAIDE score.^15^, ^59^, ^60^ Amyloid‐ and tau PET, alongside markers of vascular pathology, will help clarify the relative contributions of these differing pathologies to PI impairments. The relationship with tau pathological burden, in particular, will be clarified in follow‐on studies, building on the recent observation that CSF tau levels predicted allocentric spatial memory performance using the 4MT.^61^

Multimodal MRI using ultra high field 7T was undertaken to identify the potential neural correlates of the PI impairments. Structural MRI did not reveal any associations between PI and volumes of brain regions of interest, even at subfield level. This, however, is consistent with previous findings in presymptomatic familial AD populations^62^ and prior PREVENT imaging studies, which did not identify clear patterns of atrophy.^63^ Considering our participant age (approximately two decades away from predicted dementia onset), the absence of volumetric change may indicate an absence of regional neurodegeneration in this cohort at this early stage in the disease process. By comparison, fMRI studies revealed an association between negative hexadirectional grid‐like fMRI signal in the posterior–medial EC and PI impairment in hereditary and physiological at‐risk individuals. Further analyses aiming to understand better this negative signal revealed instead a strong unidirectional modulation of the fMRI signal. This functional imaging change may be indicative of a change in navigational strategy with increased reliance on a head direction–based approach to navigation.^64^ An overreliance on visual‐based head directional signals during the outbound triangle path, at the detriment of performing accurate distance coding, could result in an angular reproduction error during the return path^10^—which was accentuated when distal orientation cues were removed.

Limitations in our study design include the variable time‐locking between PREVENT cognitive testing and PI assessment, which we controlled for by including study visit as a nuisance regressor in regression models, and the relatively small sample size for exploring interactive risk factor effects in midlife, which warrant replication in a larger scale study with biomarkers. We also highlight the high proportion of excluded “out‐of‐bounds trials” (see Supplementary Materials), which occurred due to limitations of space available to conduct the VR assessment. This is a critical drawback of future potential use of technologies such as VR in clinical settings. Although risk factors were not associated with significantly increased rates of excluded trials, the propensity of individuals to search beyond the test area may in itself be indicative of impaired wayfinding abilities, which could be explored in future work.

In conclusion, these results indicate that impaired PI may be the initial behavioral change in AD, prior to memory decline, and as such may represent the critical point transition from at‐risk status to clinical disease onset. In addition to the benefits for clinical practice in terms of early detection and optimizing future therapeutic interventions, these discoveries using a test based on the function of EC grid cells aid translational research in delivering a platform by which studies of AD at the cellular level may be linked to understanding the onset of the clinical disorder.

## CONFLICT OF INTERESTS STATEMENT

The authors have no conflicts of interest (see supporting information).

## CONSENT STATEMENT

All participants gave written informed consent prior to their participation in the study.

## Supporting information

Supporting information

Supporting information

## Data Availability

Code is available at https://tinyurl.com/yc3ybr6e and data are available on request from PREVENT Dementia https://preventdementia.co.uk/for‐researchers/

## 1

PREVENT Dementia Collaborators: Craig Ritchie, Karen Ritchie, John O'Brien, Brian Lawlor, Lorina Naci, Graciela Muniz‐Terrera, Paresh Malhotra, Ivan Koychev, Willie Stewart, Jean Manson, Katie Wells, Sarah Gregory, Hannah Darwin, Siobhan Coleman, Zeynep Sahin, Diana Benitez‐Jimenez, Peter Gorman, Kim Tysall, David Driscoll, Gordon Wilcock, Nick Fox, Ian McKeith.
