# Supplementary material for: Entorhinal‐based path integration selectively predicts midlife risk of Alzheimer's disease
Source: Alzheimers Dement. 2024 Feb 29;20(4):2779–93. doi: 10.1002/alz.13733 (PMC11032581; doi:10.1002/alz.13733)
Supplement: Supplementary file 1 — Supporting information [file ALZ-20-2779-s001.docx]

Supplementary Text

**Out of bounds trial exclusion**

We labelled trials as ‘out of bounds’ if participants tracked 30cm beyond the 4x4m^2^ test area which resulted in a safety warning message appearing in their sightline telling them to stop walking. As the message provided a local egocentric landmark cue that could have disrupted path integration-based computations, these trials were excluded from analysis of location error. While APOE-ε4 + showed more frequent out of bounds events of borderline significance (χ^2^(1,3564) = 4.00, *p* = 0.05), FH+ or individuals with above median CAIDE score did not (both *p* > 0.16), suggesting minimal bias was introduced by excluding out of bounds trials.

We explored whether excluding out of bounds trials affected our main conclusions about overturning errors by developing a proxy measure of angular error for out of bounds trials. We used the location of the boundary collision instead of location of participant trigger pull to provide an estimate of participants’ initial heading direction for out of bounds trials. Because boundary collisions were mostly caused by under-turning (Fig. S2A), we used signed angular error in allocentric space to better account for the form of error made. This confirmed that overturning was still the predominant form of angular error event with inclusion of out of bounds trials (Fig. S2B).

We also used this all-trial signed angular error to assess if inclusion of out of bounds trials influenced our main conclusions about the effect of midlife AD risk and sex on path integration performance, which was based on regular trials only. Re-running the same analysis on all trials (namely, a multiple linear regression predicting an interaction between all risk statuses and sex on the change in signed angular error from baseline to no distal cues) showed an overall similar but weaker pattern of results (CAIDE *F*_1,78_ = 4.03, *p* = 0.048; FH x APOE-Ε4 *F*_1,78_ = 2.20, *p* = 0.142; FH x sex *F*_1,78_ = 3.86, *p* = 0.053; Fig. S3). However, because the location of boundary collision did not reflect the true final participants’ estimate of cone 1, we did not use this proxy outcome measure to draw conclusions about the data.

**Grid-like activity controls**

Hexadirectional grid-cell-like representation activity magnitude predicted path integration performance (as described in main text), but population-level grid-like representation activity magnitude across all participants was non-significant, i.e. not greater than zero (one-tail t-test *t*_52_=0.5, *p*=0.6). However, stratifying participants by above and below median path integration performance groups showed there was significantly higher grid cell representation magnitudes in the better performing group (two sample t-test *t*_47_ = 2.00, *p* = 0.04) and the better performing group trended towards significant grid activity representations (mean = 0.109; one-tail t-test *t*_26_ = 0.8, *p* = 0.200).

Temporal stability was not significantly different from chance (one-way t-test *t*_52_ = 0.04, *p* = 0.500) but there was significant spatial stability (mean Rayleigh Z = 2.38, one-way t-test *t*_52_=12.0, *p*<0.001). There was no effect of FH+ or APOE-ε4+ on temporal stability (Welch two-way t-tests, both *p* > 0.10), but there was a trend association between increased CAIDE and decreased temporal stability (Pearson’s *r* = -0.24, *p* = 0.090), in line with findings in APOE-ε4 carriers and older adults ^7,48^. Grid-cell-like activity magnitude was not related to the volume (*r*=0.012, *p*=0.930) or temporal signal-to-noise ratio (*r*=-0.042, *p*=0.760) of the posterior-medial entorhinal cortex. There was no effect of risk status on temporal signal-to-noise ratio (Welch two-sample t-test, all *p* > 0.2) or volume of the posterior-medial entorhinal cortex (all *p* > 0.3).

To confirm the specificity of the hexadirectional grid cell-like activity, we repeated the same multiple linear regressions predicting change in location error using standard controls of 4, 5 and 7-fold symmetrical models of grid cell activity. This revealed no significant associations (4-fold β = 0.04 ± 0.10, t_46_ = 0.45, *p* = 0.650; 5-fold β < 0.01 ± 0.08, t_46_ < 0.01, *p* = 0.999; 7-fold β = 0.12 ± 0.11, t_46_ = 1.12, *p* = 0.270).

**Negative hexadirectional grid-like activity**

In an exploratory analysis we examined for unidirectional modulation of the pmEC grid signal, hypothesising that it might underlie the negative hexadirectional grid signal observed in at-risk individuals with poorer PI performance. If a strong unidirectional signal is present it could interfere with the estimation of the grid angle, thereby resulting in a signal that is significant but rotated between the estimation and test data sets, hence a negative overall magnitude. Such a unidirectional signal might reflect increased activity of head-direction like processing, which aligns with the impaired angular estimation during PI without visual orientation cues in at-risk individuals. Namely, if they were over-relying on head direction signals from visual cues in the environment during the outbound path of the triangle, and therefore did not perform accurate integration of distance, it could result in angular error during the return path.^10^ To this end, we found that stronger unidirectional grid-like fMRI signals predicted an increased decline in PI with removal of distal visual orientation cues (β = 0.31 ± 0.09, *t_46_* = 3.50, *p* = 0.001; Fig. 3E).

To further explore a head-direction like effect on the signal, we examined if there was a clustering of estimated mean grid orientations of the unidirectional modulated signal in individuals using the Rayleigh test for uniformity of directions. While this was not significant for the population overall, it was significant in males only (Z = 3.34, *p* = 0.030; females Z=0.745, *p* = 0.479), in line with the apparent stronger association of unidirectional signal and PI impairments observed in males (Fig. 3F). The average mean orientation was 181º (Fig. S4), consistent with the use of a single salient landmark visible on a room wall during the fMRI task which was identical across all participants. Future work is needed to further understand different behavioural mechanisms during navigation and to confirm the association with AD risk factors.

Author contributions:

Conceptualization: DC

Methodology: CN, DC, MP, RH, ZJ, MS, MED, TW, NB

Software: MS, NB, AC, TW

Investigation: CN, MP, CR, CTR

Visualization: CN, ZJ

Funding acquisition: DC, CWR

Project administration: CN, JOB, LS, IK, PM, KR, CWR

Supervision: DC, JOB, LS

Writing – original draft: CN, DC

Writing – review & editing: All


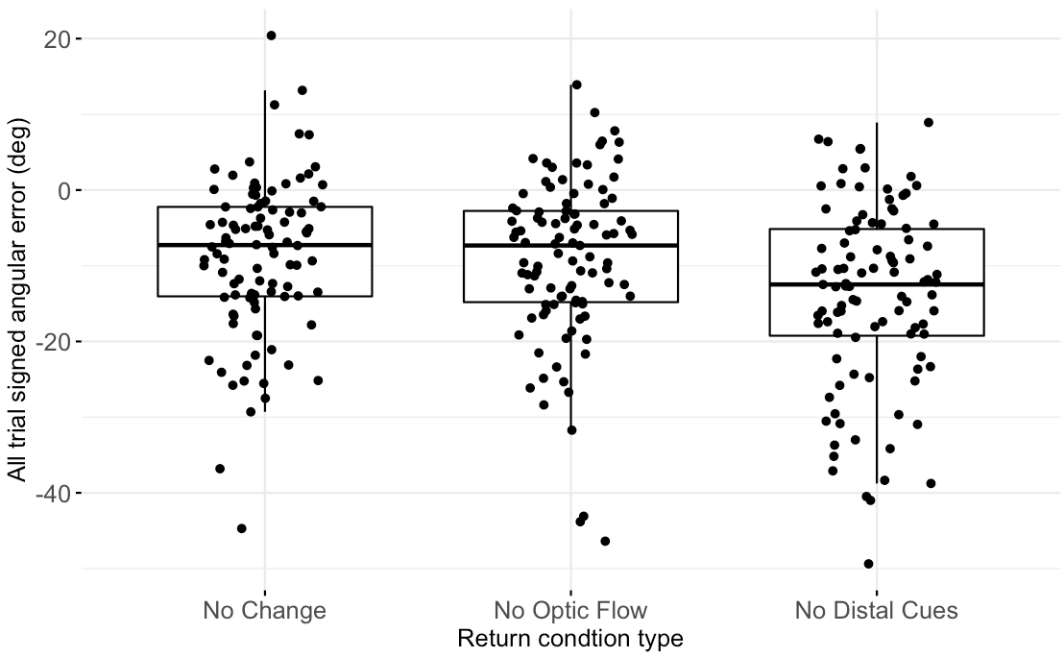
Supplementary Figures

**Figure S1.** Change in allocentric signed angular error from baseline to “no distal cues” conditions**.** All participants showed negative angular errors, corresponding to over-turning, which was magnified when distal cues were removed.

**Figure S2.** Allocentric signed angular error for regular trials and out of bounds trials.


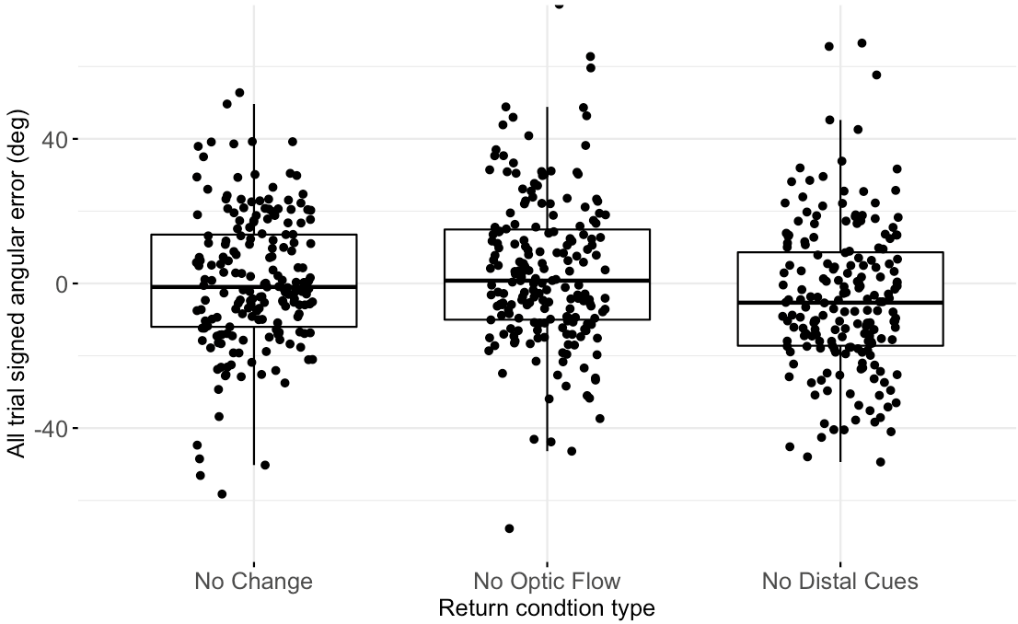

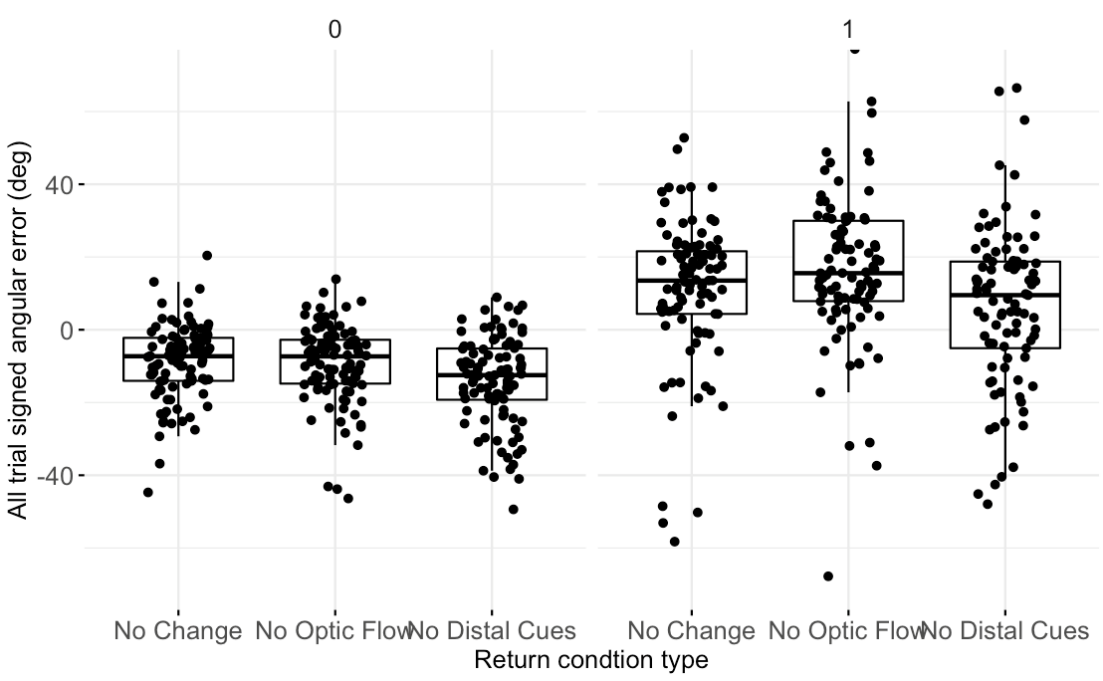


A

B

Regular trials

Out of bounds trials

(**A**) Out of bounds trials associated with more under-turning (positive angular errors) than over-turning (negative values). (**B**) Overall across all trials, both regular and out of bounds, the angular errors (especially for the no distal cues trials) were still driven by over-turning even with out of bounds trials accounted for.

**Figure S3.** Replicating main effects after inclusion of out of bounds trials with proxy angular error measure. (**A**): male FH+/APOE-ε4+ visually trend towards larger over-turns across all trials from baseline to no distal cues condition, recapitulating the main effects seen for regular trials only. (**B**): higher CAIDE associates with greater over-turning errors from baseline to no distal cues conditions. Negative values indicate increases in over-turning relative to cone 1; positive values increases in under-turning.


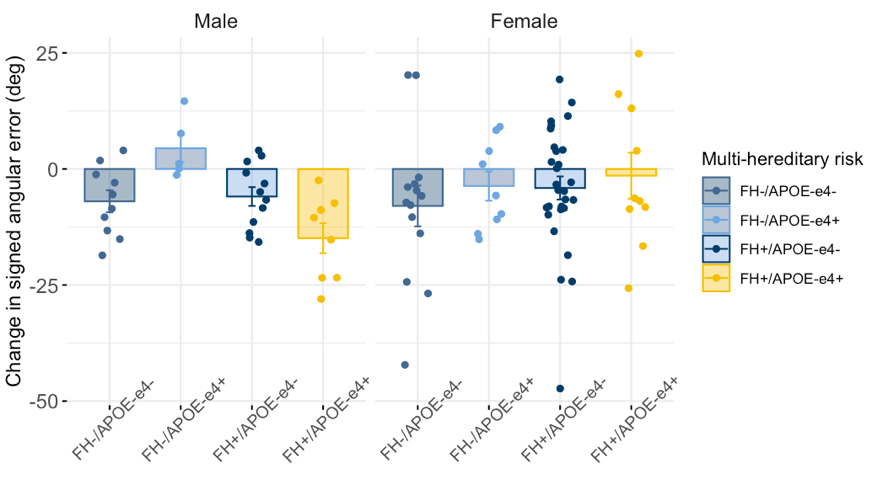

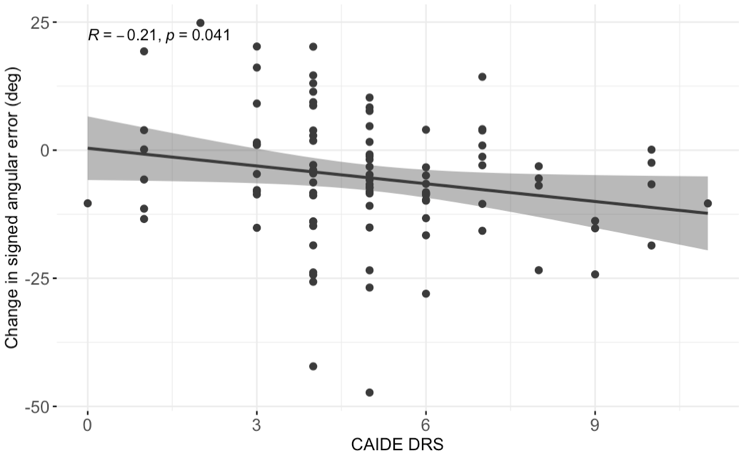


A

B


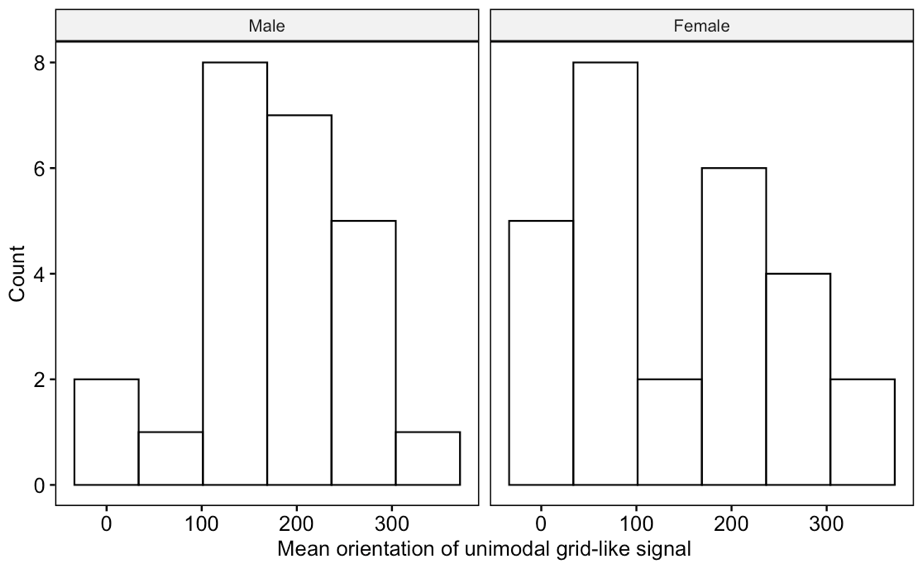


**Figure S4.** Mean estimated grid orientations of the unidirectional signal are significantly clustered in males only. Left: male individuals show a clustering of estimated mean orientations around 181º, whereas females show uniformly distributed orientations (right).

Supplementary Tables

| Table S1 Demographics for main sample, stratified by sex | | | |
| --- | --- | --- | --- |
| Characteristic | **Male**  N = 35 | **Female**  N = 64 | ***p*** |
| Age |  |  |  |
| Mean yrs | 57.6 ± 5.23 | 56.0 ± 5.27 | 0.20 |
| Education |  |  |  |
| Mean yrs | 16.1 ± 2.42 | 17.4 ± 3.22 | 0.05* |
| APOE-ε4  Positive (%)  NA (%) | 13 (37%)  0 | 19 (29%)  2 (3%) | 0.70^a^ |
| Family history ^†^  Positive (%) | 20 (57%) | 41 (64%) | 0.70^a^ |
| ^a^ Pearson Chi-square test  ^b^ Wilcoxon rank sum test  ^†^ parental | | | |

| Table S2 Demographics for MRI subgroup, stratified either by family history or APOE-ε4 status | | | | | | | | |
| --- | --- | --- | --- | --- | --- | --- | --- | --- |
| Characteristic | **Family history positive**  N = 33 | **Family history negative**  N = 21 | ***p*** | **APOE-ε4 positive**  N = 18 | **APOE-ε4 negative**  N = 35 | ***p*** | **Whole sample**  N=54 |  |
| Sex |  |  |  |  |  |  |  |  |
| Female (%) | 18 (53%) | 12 (57%) | *0.70^a^* | 11 (61%) | 18 (50%) | *0.60^a^* | 30 (56%) |  |
| Age |  |  |  |  |  |  |  |  |
| Mean yrs (SD) | 57.2 ± 5.10 | 56.2 ± 4.33 | *0.37* | 55.9 ± 4.71 | 57.2 ± 4.87 | *0.40^b^* | 56.8 ± 4.80 |  |
| Education |  |  |  |  |  |  |  |  |
| Mean yrs (SD) | 16.6 ± 3.09 | 16.5 ± 3.06 | *0.92* | 16.7 ± 3.06 | 16.6 ± 2.98 | *1^b^* | 16.5 ± 3.05 |  |
| APOE-ε4  Positive (%)  NA (%) | 12 (36%)  1 (3%) | 6 (28%)  0 | *0.80^a^* | -  - | -  - |  | 18 (33%)  1 (2%) |  |
| Family history ^†^  Positive (%) | - | - |  | 12 (67%) | 20 (58%) | *0.70^a^* | 33 (61%) |  |
| Family history type  Maternal (%)  Paternal (%)  Both (%) | 13 (39%)  22 (67%)  2 (6%) | -  -  - |  | 7 (39%)  7 (39%)  2 (11%) | 5 (17%)  15 (44%)  0 | *0.09^a^*  *1.00^a^*  *0.50^a^* | -  -  - |  |
| CAIDE  Mean score (SD)  NA (%) | 5.71 ± 2.21  2 (6%) | 5.05 ± 2.31  0 | *0.20^b^* | 5.61 ± 2.34  0 | 5.35 ± 2.37  1 (3%) | *0.80^b^* | 5.44 ± 2.25  2 (4%) |  |
| ^a^ Pearson Chi-square test  ^b^ Wilcoxon rank sum test  ^†^ parental  CAIDE = Cardiovascular risk factors, Ageing and Dementia Incidence Study. | | | | | | | | |

| Table S3 Demographics for combined family history and APOE-ε4 risk groups | | | | | |
| --- | --- | --- | --- | --- | --- |
| Characteristic | **FH+/APOE-ε4+** N = 18 | **FH+/APOE-ε4-**  N = 41 | **FH-/APOE-ε4+**  N = 14 | **FH-/APOE-ε4-**  N = 24 | ***p*** |
| Age |  |  |  |  |  |
| Mean yrs (SD) | 56.1 ± 6.02 | 57.5 ± 4.82 | 54.1 ± 4.82 | 56.8 ± 5.73 | *0.23 ^a^* |
| Sex |  |  |  |  |  |
| Female (%) | 10 (56%) | 29 (71%) | 9 (64%) | 14 (58%) | *0.60 ^b^* |
| Education |  |  |  |  |  |
| Mean yrs (SD) | 16.7 ± 3.05 | 17.1 ± 2.91 | 17.0 ± 2.72 | 16.6 ± 2.99 | *0.90 ^a^* |
| CAIDE  Mean score (SD) | 5.28 ± 2.44 | 5.05 ± 2.11 | 4.50 ± 2.18 | 5.08 ± 2.28 | *0.88 ^a^* |
| ^a^ ANOVA  ^b^ Pearson Chi-square test  ^†^ parental | | | | | |

| Table S4 ANOVA nested model comparison of predictors for change in location error from baseline to no distal cues conditions | |
| --- | --- |
| Predictors | **Adjusted R^2^** |
| FH x APOE x CAIDE x sex | 0.26 |
| FH x APOE x sex | 0.20 |
| FH x APOE x CAIDE | 0.13^a^ |
| FH x sex | 0.11^a^ |
| APOE x sex | 0.07^a^ |
| CAIDE x sex | 0.05^a^ |
| All models controlled for age and education, and sex where not already included  ^a^ showed significantly lower adjusted R^2^ than full risk factor x sex model  FH = family history  APOE = APOE-ε4 allele  CAIDE = Cardiovascular Risk Factors, Aging and Dementia Study dementia risk score | |

| Table S5 Effect of AD risk factors on comparator task performance | | | | | | |
| --- | --- | --- | --- | --- | --- | --- |
| Risk Factor | **4MT** | **VRSTT** | **VSTBT** | **Narrative Recall** | **Name-Face** | **ACE-R** |
| FH+ | 10.1 ± 0.3 | 9.8 ± 0.2 | 0.73 ± 0.03 | 13.6 ± 0.6 | 5.4 ± 0.2 | 96.7 ± 0.4 |
| FH- | 11.4 ± 0.4 | 10.1 ± 0.3 | 0.71 ± 0.03 | 12.6 ± 0.7 | 5.5 ± 0.3 | 96.4 ± 0.5 |
| *p_FH_* | ***0.016*** | *ns* | *ns* | *ns* | *ns* | *ns* |
| *p_FH x sex_* | *ns* | ***0.008 ^b^*** | *ns* | *ns* | *ns* | *ns* |
| APOE-ε4 + | 10.6 ± 0.3 | 10.3 ± 0.3 | 0.69 ± 0.03 | 13.3 ± 0.8 | 5.15 ± 0.3 | 96.9 ± 0.5 |
| APOE-ε4 - | 10.7 ± 0.5 | 9.6 ± 0.2 | 0.74 ± 0.02 | 13.2 ± 0.6 | 5.55 ± 0.2 | 96.5 ± 0.4 |
| *p_APOE_* | *ns* | *ns* | *ns* | *ns* | *ns* | *ns* |
| *p_APOE-Ε4 x sex_* | *ns* | *ns* | *ns* | *ns* | *ns* | *ns* |
| CAIDE β | -0.21 ± 0.18 | 0.07 ^C^ | -0.01 ± 0.01 | 0.11 ± 0.31 | -0.39 ± 0.09 | -0.07 ^c^ |
| *p* | *ns* | *ns* | *ns* | *ns* | ***< 0.001*** | *ns* |
| FH+/APOE-ε4 + | 9.7 ± 0.6 | 10.2 ± 0.4 | 0.69 ± 0.04 | 14.2 ± 1.0 | 5.27 ± 0.4 | 97.3 ± 0.4 |
| FH-/APOE-ε4 + | 12.0 ± 0.6 | 10.5 ± 0.5 | 0.70 ± 0.05 | 12.4 ± 1.2 | 5.01 ± 0.5 | 96.3 ± 0.8 |
| FH+/APOE-ε4 - | 10.1 ± 0.5 | 9.3 ± 0.4 | 0.75 ± 0.03 | 13.3 ± 0.7 | 5.34 ± 0.3 | 96.2 ± 0.5 |
| FH-/APOE-ε4 - | 11.2 ± 0.5 | 10.1 ± 0.4 | 0.71 ± 0.04 | 12.9 ± 0.9 | 5.84 ± 0.4 | 96.5 ± 0.6 |
| *p_Multifactor status_* | ***0.019 ^a^*** | *ns* | *ns* | *ns* | *ns* | *ns* |
| *p* values corrected for age, sex, and education in multiple linear regression per individual risk factor  ^a^ driven by singular difference in FH+/APOE-ε4 + vs FH-/APOE-ε4 + (*p_Tukey_* = 0.058)  ^b^ driven by difference in FH+ vs FH- in females (*p* = 0.006)  ^c^ Spearman’s Rho shown given ceiling effects in the data  FH = family history  CAIDE = Cardiovascular risk factors, Ageing and Dementia Incidence Study Dementia Risk Score | | | | | | |

| Table S6 Multivariate AD risk factor differences on relative ROI volumes (% of total intracranial volume) | | | | | | | | | | | | |
| --- | --- | --- | --- | --- | --- | --- | --- | --- | --- | --- | --- | --- |
| Risk Factor | **CA1** | **CA2** | **CA3** | **DG** | **Sub** | **pmEC** | **alEC** | **PrC** | **PhC** | **RSC** | **PCC** |  |
| FH+ | 0.142 | 0.006 | 0.029 | 0.101 | 0.206 | 0.089 | 0.094 | 0.106 | 0.092 | 0.476 | 0.567 |  |
| FH- | 0.146 | 0.007 | 0.029 | 0.100 | 0.206 | 0.082 | 0.096 | 0.103 | 0.100 | 0.479 | 0.584 |  |
| *p* | *0.35* | *0.14* | *0.84* | *0.68* | *0.72* | *0.38* | *0.57* | *0.78* | *0.11* | *0.88* | *0.32* |  |
| APOE-ε4 + | 0.138 | 0.006 | 0.028 | 0.095 | 0.204 | 0.089 | 0.092 | 0.107 | 0.094 | 0.478 | 0.564 |  |
| APOE-ε4 - | 0.147 | 0.007 | 0.029 | 0.103 | 0.207 | 0.084 | 0.097 | 0.104 | 0.096 | 0.477 | 0.579 |  |
| *p* | *0.11* | ***0.04*** | *0.50* | ***0.02*** | *0.75* | *0.47* | *0.66* | *0.33* | *0.58* | *0.88* | *0.59* |  |
| CAIDE β | < -.001 | < -.001 | < -.001 | < -.001 | 0.003 | < -.001 | < -.001 | < -.001 | < .001 | .007 | < .001 |  |
| *p* | *0.68* | ***0.05*** | *0.16* | *0.88* | ***0.02*** | *0.69* | *0.93* | *0.31* | *0.64* | ***0.08*** | *0.96* |  |
| *p* values corrected for age, sex, and education but not multiple comparisons. False discovery rate corrected values all *p_FDR_* > 0.183  β regression values for CAIDE predictor  FH = family history; CAIDE = Cardiovascular risk factors, Ageing and Dementia Incidence Study Dementia Risk Score; CA = cornu ammonis, DG = detate gyrus, Sub = subiculum, pmEC = posterior-medial entorhinal cortex, alEC = anterior-lateral entorhinal cortex, PrC = perirhinal cortex, PhC = parahippocamapal cortex, RSC = retrosplenial cortex, PCC = postering cingulate cortex. | | | | | | | | | | | | |
